# Supplementary material for: Physical Activity Dynamically Regulates the Hippocampal Proteome along the Dorso-Ventral Axis
Source: Int J Mol Sci. 2020 May 15;21(10):3501. doi: 10.3390/ijms21103501 (PMC7278950; doi:10.3390/ijms21103501)

Supplementary Figure S2

QUANTITATIVE MASS SPECTROMETRY ANALYSIS

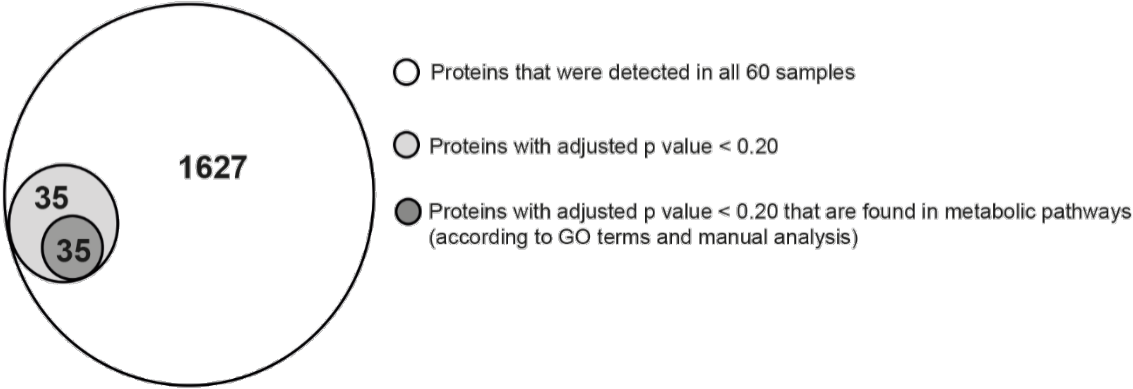

Supplement: Supplementary file 1 [file ijms-21-03501-s001.zip › Supplementary Figures S2 related to Figure 3.pdf]
