# Supplementary figures and images for: Physical Activity Dynamically Regulates the Hippocampal Proteome along the Dorso-Ventral Axis
Source: Int J Mol Sci. 2020 May 15;21(10):3501. doi: 10.3390/ijms21103501 (PMC7278950; doi:10.3390/ijms21103501)

**Dorsal Hippocampus**

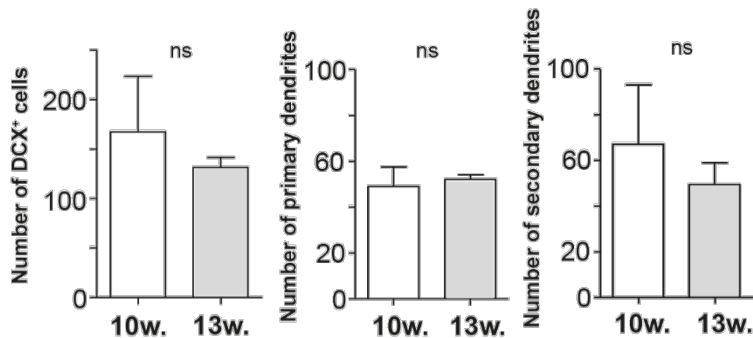

**Ventral Hippocampus**

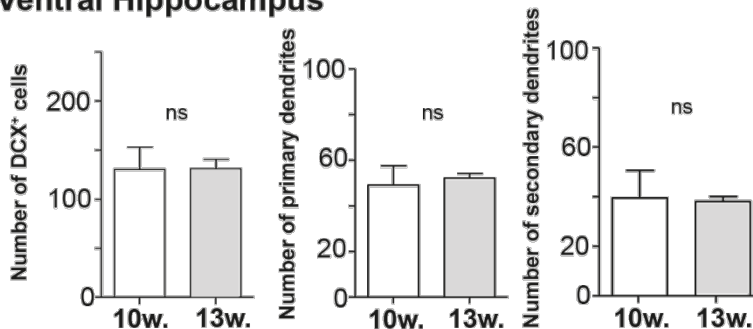

Supplement: Supplementary file 1 [file ijms-21-03501-s001.zip › Supplementary Figures S1 related to Figure 1.pdf]

## Dorsal hippocampus

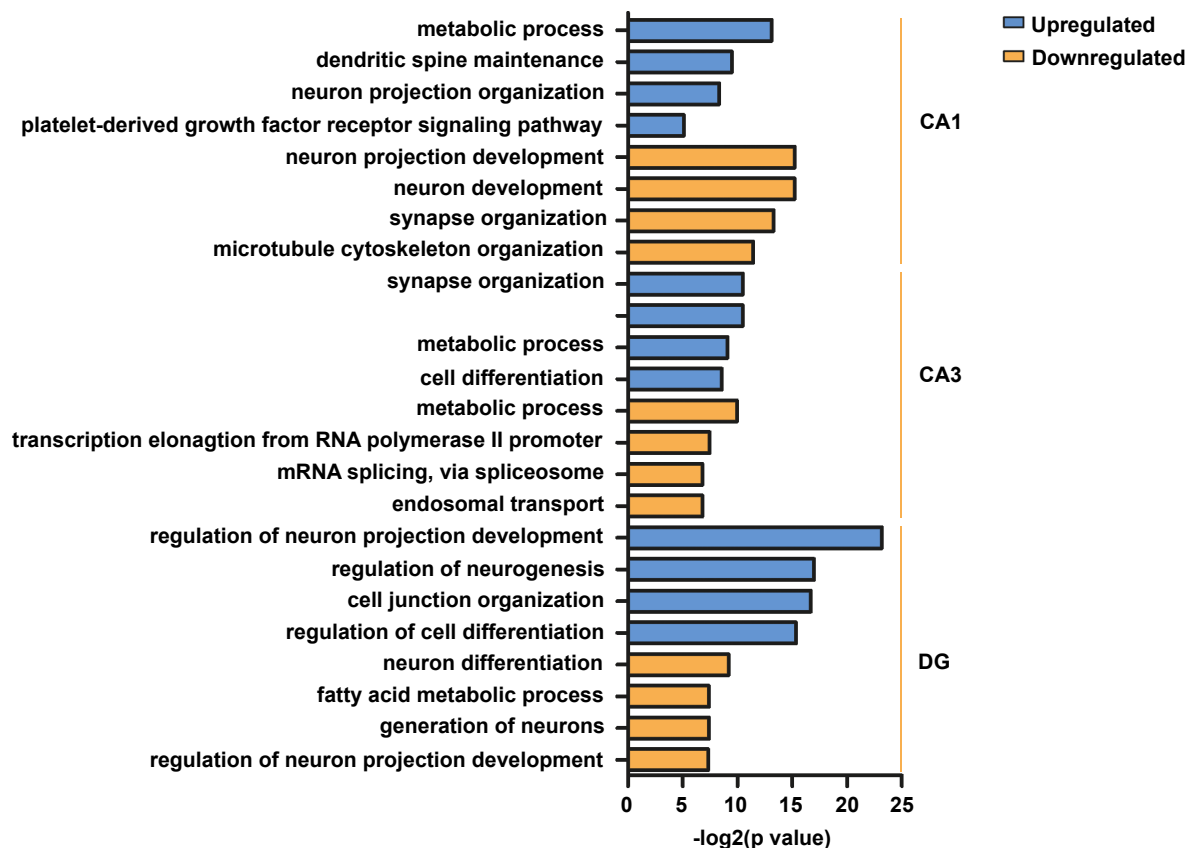

## Ventral hippocampus

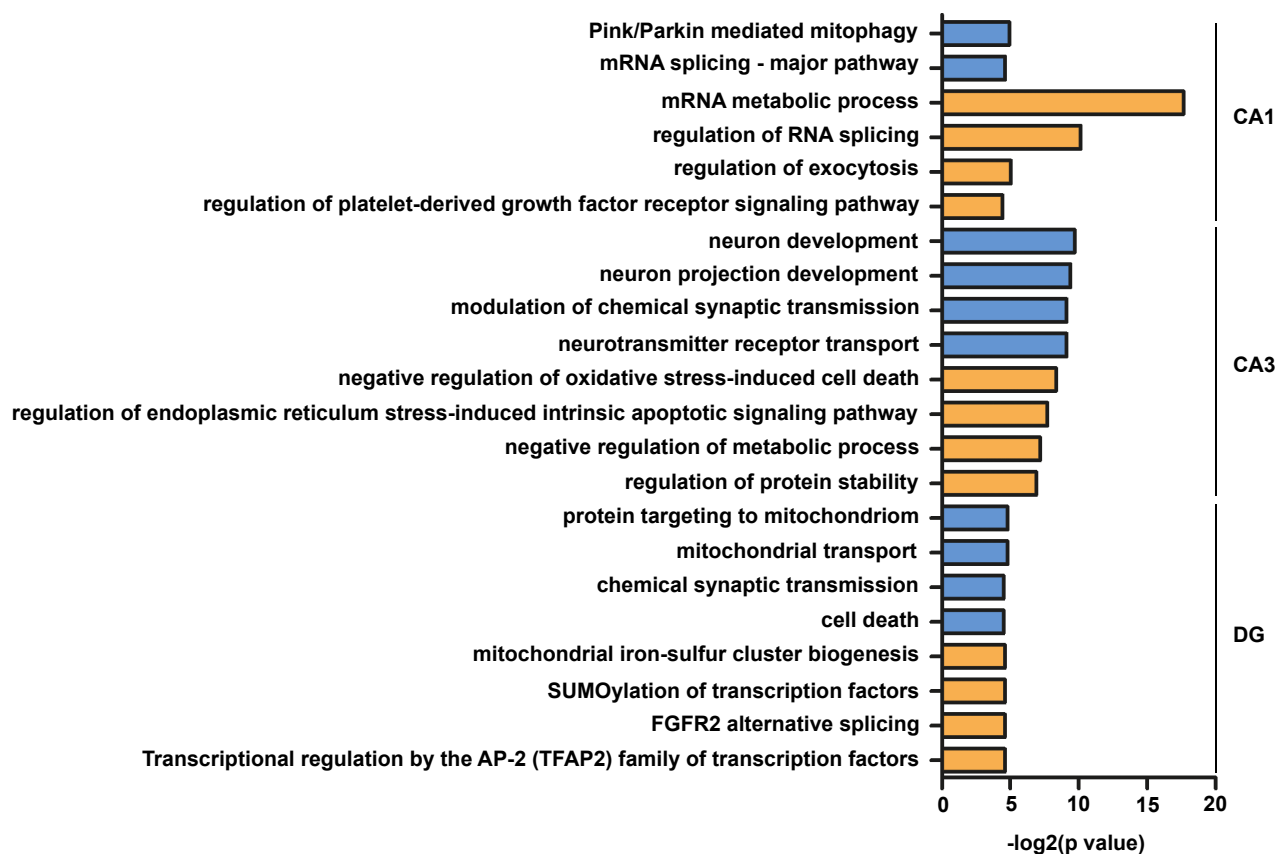

Supplement: Supplementary file 1 [file ijms-21-03501-s001.zip › Supplementary Figures S3 related to Figure 4and5.pdf]
